# Phytoremediation of Soil Contaminated by Organochlorine Pesticides and Toxic Trace Elements: Prospects and Limitations of *Paulownia tomentosa*

**DOI:** 10.3390/toxics10080465

**Published:** 2022-08-11

**Authors:** Aigerim Mamirova, Almagul Baubekova, Valentina Pidlisnyuk, Elvira Shadenova, Leyla Djansugurova, Stefan Jurjanz

**Affiliations:** 1 Department of the Environmental Chemistry & Technology, Faculty of the Environment, Jan Evangelista Purkyně University, Pasteurova 15, 400 96 Usti nad Labem, Czech Republic; 2 Institute of Genetics and Physiology SC MES RK, Al-Farabi 93, Almaty 050060, Kazakhstan; 3 Department of Biotechnology, Faculty of Biology and Biotechnology, Al-Farabi Kazakh National University, Al-Farabi 71, Almaty 050040, Kazakhstan; 4 Unité de Recherches—Animal et Fonctionnalités des Produits Animaux, Université de Lorraine-INRAE, 54000 Nancy, France

**Keywords:** *Paulownia tomentosa*, phytoremediation, organochlorine pesticides, toxic trace elements, bioconcentration factor, translocation factor

## Abstract

*Paulownia tomentosa* (Thunb.) Steud is a drought-resistant, low-maintenance and fast-growing energy crop that can withstand a wide range of climatic conditions, provides a high biomass yield (approximately 50 t DM ha^−1^ yr^−1^), and develops successfully in contaminated sites. In Kazakhstan, there are many historically contaminated sites polluted by a mixture of xenobiotics of organic and inorganic origin that need to be revitalised. Pilot-scale research evaluated the potential of *P. tomentosa* for the phytoremediation of soils historically contaminated with organochlorine pesticides (OCPs) and toxic trace elements (TTEs) to minimise their impact on the environment. Targeted soils from the obsolete pesticide stockpiles located in three villages of Talgar district, Almaty region, Kazakhstan, i.e., Amangeldy (soil A), Beskainar (soil B), and Kyzylkairat (soil K), were subjected to research. Twenty OCPs and eight TTEs (As, Cr, Co, Ni, Cu, Zn, Cd, and Pb) were detected in the soils. The phytoremediation potential of *P. tomentosa* was investigated for OCPs whose concentrations in the soils were significantly different (aldrin, endosulfans, endrin aldehyde, HCB, heptachlor, hexabromobenzene, keltan, methoxychlor, and γ-HCH) and for TTEs (Cu, Zn, and Cd) whose concentrations exceeded maximum permissible concentrations. Bioconcentration (*BCF*) and translocation (*TLF*) factors were used as indicators of the phytoremediation process. It was ensured that the uptake and translocation of contaminants by *P. tomentosa* was highly variable and depended on their properties and concentrations in soil. Besides the ability to bioconcentrate Cr, Ni, and Cu, *P. tomentosa* demonstrated very encouraging results in the accumulation of endosulfans, keltan, and methoxychlor and the phytoextraction of γ-HCH (*TLF*s of 1.9–9.9) and HCB (*BCF*s of 197–571). The results of the pilot trials support the need to further investigate the potential of *P. tomentosa* for phytoremediation on a field scale.

## 1. Introduction

Emissions from the oil and gas industry and mining and smelting activities are sources of various hotspots [1,2,3,4] that negatively affect the surrounding soils and surface and groundwaters. At such contaminated sites, xenobiotics of organic and inorganic origin occur simultaneously, requiring the development of innovative management strategies to address these complex issues. Only a limited number of studies have proposed solutions for addressing these complex ecological problems [1,2,3], and phytoremediation is among the proposed approaches [5,6,7].

Phytoremediation is based on the utilisation of different plants for soils contaminated by toxic trace elements (TTEs), hydrocarbons, pesticides, oil products, and radionuclides [8,9,10,11,12,13]. The plants used in phytoremediation must grow easily and quickly, and produce enough biomass to eliminate notable amounts of contaminants from the soil. Undoubtedly, the nature of remediation depends directly on the ability of the plant to uptake and accumulate the concerned pollutants. In the case of energy crops proposed as phytoagents [14,15], one of the crucial characteristics is the high content of fibre fractions (i.e., lignin, cellulose, and lignocellulose), which corresponds to the quality of the bioproducts to be produced.

Gołąb-Bogacz et al. indicated [16] *Miscanthus* spp. as the most promising plant suitable for phytoremediation. These species have a high annual biomass yield (up to 36.6 t ha^−1^) [17] and the highest heating value of 16.3–20.2 MJ kg^−1^ [18]. Another prospective energy crop, *Arundo donax*, has been shown to produce even higher biomass yields than *M. × giganteus*, as it can be harvested several times a year [19]. However, considering the energy costs, *M. × giganteus* remains a more promising plant as it requires fewer inputs during cultivation [20,21]. Numerous studies have demonstrated the efficacy of *M. × giganteus* and *A. donax* in the phytoremediation of TTE-contaminated soils [12,22,23,24,25,26]; however, when the soil was contaminated with organochlorine pesticides (OCPs), *M. × giganteus* could not tolerate their high concentrations and survived in the soil, overcoming the maximum permissible concentration (MPC) for Kazakhstan (241 ± 16 µg kg^−1^) in two instances only [27], while studies investigating the remediation potential of *A. donax* with respect to OCPs were not found. Indeed, *M. sinensis*, another representative of *Miscanthus* spp., showed good growth in OCP-contaminated soil, with concentrations up to 62 × MPC [27]. However, the cultivation of *M. sinensis* and *A. donax* is restricted in some countries due to their invasiveness [28,29], which shortens the list of plants eligible for the phytoremediation of soils contaminated with OCPs and TTEs and necessitates the search for new plants that can be used.

In this context, *Paulownia tomentosa* (Thunb.) Steud [30] can be considered a good candidate for the phytoremediation of soil contaminated with a mixture of OCPs and TTEs. The plant can produce consistent biomass of 50 t DM ha^−1^ y^−1^ under non-optimised growth conditions and up to 330 t DM ha^−1^ y^−1^ under optimised ones [30,31], which is around two times higher compared to other popular woody plants: willow (23.1–25.7 t DM ha^−1^) and poplar (12.2 ± 0.3 t DM ha^−1^) [32,33]. Moreover, *P. tomentosa* is a drought-resistant, low-maintenance plant able to withstand a wide range of climatic conditions (i.e., from −20 to +40 °C) [34]. The biomass content of *P. tomentosa* is similar to that of *Miscanthus* sp., i.e., 22.4% lignin, 37.4% cellulose, 33.3% hemicellulose, and 61.5–70.7% holocellulose [30,35], which makes its biomass promising for processing into various bioproducts. The published research on the application of *P. tomentosa* is mainly concerned with TTE-contaminated soils [36,37,38,39,40]; there are also a few reports on the use of the plant in PCB- and hydrocarbon-contaminated soils [3], while this plant’s tolerance to OCP-contaminated soils is not presented in the literature, although complex contaminated soils are common in the territories of former obsolete pesticide stockpiles [41], mining [42], and post-military soils [27].

The current study aimed to investigate the phytoremediation efficiency of *P. tomentosa* toward soil historically contaminated by a mixture of OCPs and TTEs and evaluate the interconnections between the background of the aged soils and the plant phytoremediation potential. The use of historically contaminated soils allowed us to conduct experiments in the conditions of the natural heterogeneity of the compounds’ distribution in the soils.

## 2. Materials and Methods

### 2.1. Soil Collection

The research soils were sampled on April 2018 at historically contaminated sites in the vicinity of obsolete pesticide storage facilities located in three villages of Talgar district, Almaty region, Kazakhstan: (a) Amangeldy (GPS 43°18′01.54″ N, 77°12′33.9″ E), hereafter denoted as soil A; (b) Beskainar (GPS 43°13′16″ N, 77°6′49″ E), soil B; and (c) Kyzylkairat (GPS 43°17′58.7″ N, 77°11′39.6″ E), soil K. According to the updated Köppen–Geiger classification [43], the climate of the Talgar region belongs to group *Dfa*: it has cold winters, hot summers, and does not have a dry season.

Soil sampling was done according to the standard procedure (ISO 18400-203:2018) [44]: five samples were collected from a 5 × 5 m testing square at a depth of 0–0.6 m. After sampling, plant materials and stones were manually removed; soil was further air-dried until constant weight and sieved (d = 3 mm). According to the World Reference Base for Soil Resources Classification, the soils at the three research sites belonged to kastanozems [45].

The agrochemical parameters of the research soils were determined using standard methods. The total humus content (C) was measured by the Tyurin method [46]; the content of phosphorus (P_2_O_5_) and potassium (K_2_O) mobile forms by the Kirsanov method with the modification of the Central Research Institute for Agrochemical Agricultural Services [47]; the absorbed bases of sodium (Na) and potassium (K) by the Antipov-Karataev and Mametov method with Grabarov modification; the absorbed bases of calcium (Ca) and magnesium (Mg) by the Arinushkin method with Grabarov modification; and the soil pH according to GOST 26423-85 [48]. The agrochemical parameters of the research soils are presented in Table 1. These soils were used in the pot experiment performed in greenhouse conditions.

### 2.2. Experiment Design

The clones of *P. tomentosa* were first obtained by in vitro propagation and further adapted to the open ground conditions. The adaptation took place for three months (January–March 2019), when the clones were illuminated with fluorescent lamps LB-40-4 of the infrared spectrum at 3000 lux. After the adaptation period, *P. tomentosa* seedlings were planted in pots, and each pot was filled with 350 g of dried research soil. The surface area of one pot was 88.56 cm^2^; consequently, the total area was 0.74 m^2^. Overall, 84 seedlings were planted, 28 plants per research soil. The experiment was started on 18 March 2019 and ended on 15 June 2020. Soil moisture was adjusted to 50% by irrigation with regular cold tap water every 3rd day.

### 2.3. Biomass Collection at Harvest

Research plants were harvested on 15 June 2020. The plant’s roots and aboveground biomass (AGB) were sampled following GOST 17.4.4.02-84 [49]. Root samples were taken by unearthing the plant together with the soil from the pot. The roots were shaken free of the soil and washed thoroughly under cold running tap water to eliminate tiny soil particles and then oven-dried till constant weight. Plants’ AGB was dried in the same way as roots. The samples were separately collected in labelled plastic zip-lock bags and then stored at room temperature until the chemical analysis.

### 2.4. Chemical Analysis

Concentrations of OCPs were measured by gas chromatography with an electron capture detector (Gas Chromatography 6890N Agilent Technologies, Santa Clara, CA, USA) equipped with the autosampler Combi-PAL (CTC Analytics AG, Zwingen, Switzerland). Limit of detection (LOD) values for soil and plant samples were 0.1 and 25 µg kg^−1^, while limit of quantification (LOQ) values were 4.0 and 5.0 µg kg^−1^, respectively. Quartz sand and cellulose were used as reference samples according to standards ST RK 2131-2011 [50] and ST RK 2011-2010 [51], used to analyse soil and plant samples, respectively. The TTE concentrations were determined by atomic absorption spectrometry with electrothermal atomisation, using a Varian AA240 Atomic Absorption Spectrometer GTA 120 (Agilent Technologies, Santa Clara, CA, USA). The reference samples were the same as for OCP content, while LOD and LOQ values were 0.1 and 2.0 mg kg^−1^, respectively. The procedure was described in detail earlier [52]; briefly, analysis of soil samples was provided according to standards ST RK ISO 11047-2008 [53] and GOST 23581.8-79 [54]; analysis of plant samples was performed following ST RK ISO 11047-2008 [53], GOST 23581.8-79 [54], GOST 26930-86 [55], and GOST 30178-96 [56].

### 2.5. Calculation of Phytoremediation Coefficients

The bioconcentration factor (*BCF*) is the ratio between the pollutant concentration in the plant tissue and its concentration in the soil. The coefficient was calculated according to Zayed et al. [57]:

(1) $$BCF=\frac{Contaminant\;concentration\;in\;plant\;tissues\;\left(mg\;kg^{-1}\right)\;at\;harvest}{Initial\;contaminant\;concentration\;in\;soil\;\left(mg\;kg^{-1}\right)}$$

The transfer of pollutants within the plant was quantified by the translocation factor (*TLF*), which is the ratio between the pollutant’s concentration in the aboveground biomass (AGB) (leaves and stems) and root system [58]:

(2) $$TLF=\frac{Contaminant\;concentration\;in\;aboveground\;biomass\;\left(mg\;kg^{-1}\right)}{Contaminant\;concentration\;in\;roots\;\left(mg\;kg^{-1}\right)}$$

### 2.6. Statistical Analysis

Data analysis was performed using RStudio software (version 1.3.959, R Studio PBC, 2020). A one-way ANOVA was performed to compare the initial concentrations of contaminants in the soils at three research sites, while a two-way ANOVA was applied to compare the contaminant concentrations in the AGB and roots of the plants grown in the different soils. In the case of TTEs, to attain statistical and biological differences, concentrations of these substances were measured in plant tissues, referring to the soils with the highest and lowest concentrations for each particular element. The comparison of *BCF* and *TLF* values was carried out using two-way ANOVA.

Tukey HSD tests were performed for pairwise comparison of means when ANOVA showed a significant effect of the tested factors. Then, treatments were categorised by letter in descending order, and boxplots/graphs were generated. Significance was declared at *p* < 0.05; however, tendencies at *p* < 0.10 were indicated as well.

## 3. Results

### 3.1. Contamination of the Research Soils

The levels of contamination of the research soils A, B, and K by OCPs are presented in Table 2. Altogether, twenty pesticides were detected in the soils, including fourteen insecticides, three metabolites, three fungicides, and one herbicide. The concentrations of TTEs in the research soils are presented in Table 2. Due to the natural heterogeneity of the distribution of organic compounds in the complex soil matrix, the OCP contamination appeared more heterogeneous than the distribution of TTEs. To address this heterogeneity, the phytoremediation process was examined for OCPs whose concentrations in the soils were significantly different (*p* < 0.05), i.e., aldrin, endosulfans, endrin aldehyde, heptachlor, hexabromobenzene, methoxychlor, and lindane (γ-HCH), or tended to be different (i.e., *p* < 0.1), i.e., HCB and keltan.

Among the three research soils, soil K appeared to be the most contaminated with OCPs, followed by soils B and A, while contamination with TTEs showed the opposite tendency: soil A was the most contaminated, followed by soils B and K. Concentrations of aldrin and hexabromobenzene exceeded the MPC in research soils—specifically, aldrin by 4.9, 38.4, and 138 times, and hexabromobenzene by 1.3, 6.3, and 20.1 times in soils A, B, and K, respectively. Endosulfan concentrations slightly exceeded the MPC in soil B (by 1.2 times), while the exceedance in soil K was around 7.6 times. Heptachlor was detected only in soils B and K at concentrations exceeding the MPC by 2.4 and 5.4 times, respectively. The concentrations of methoxychlor and γ-HCH varied considerably between soils; however, they did not exceed the MPC in any of them. The concentration of HCH isomers exceeded the MPC in all three soils, consequently, by 1.6, 2.6, and 6.0 times in soils A, B, and K, respectively (Table 2).

The concentrations of TTEs in the research soils varied essentially (Table 2); however, MPC values were surpassed for Cu, Zn, and Cd only. Specifically, the Zn concentration in soil A was 1.6 times higher than the MPC, and the Cu concentration was higher than the MPC by 1.6, 1.4, and 1.4 times in soils A, B, and K, respectively. The highest exceedance was recorded for Cd, which exceeded the MPC by 4.3, 2.3, and 1.7 times for soils A, B and K, respectively.

### 3.2. Phytoremediation Potential of P. tomentosa Utilised in Complex OCP- and TTE-Contaminated Soils

To assess the potential of *P. tomentosa* to uptake the contaminants from the research soils, the phytoremediation coefficients *BCF* and *TLF* were calculated (Table 3). The patterns of transfer of two OCPs in the soil–plant system did not allow further analysis: heptachlor was detected only in the AGB (*BCF* of 2.8), while hexabromobenzene only in the roots (*BCF* of 2.0) of plants grown in soil B. Statistical analysis of OCP concentrations in plant tissues showed the impact of soil contamination on the uptake of endosulfans and endrin aldehyde. Similarly, the cumulative influence of contaminants’ concentrations in soils and the accumulation organs of plants was detected for HCB, keltan, methoxychlor, and γ-HCH (Table 3).

**Figure 1:**
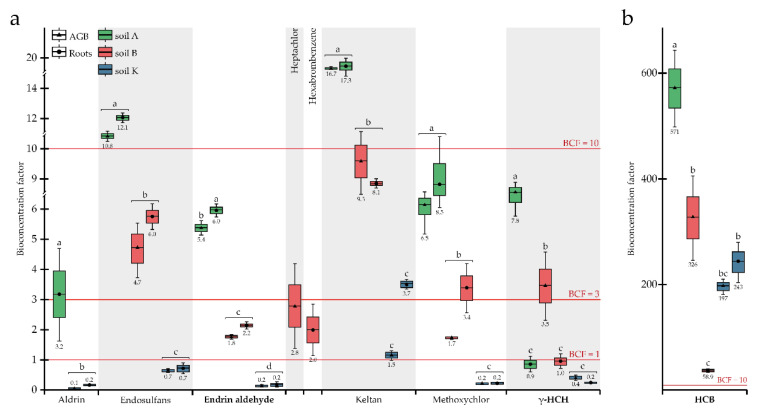
Bioconcentration factors for OCPs: (**a**) *BCF*s < 20; (**b**) *BCF*s > 50. OCPs highlighted in bold indicate a significant difference between *BCF*s due to soil origin and plant part effects. Different letters on the boxplots within one compound indicate a significant difference at *p* < 0.05.

**Figure 2:**
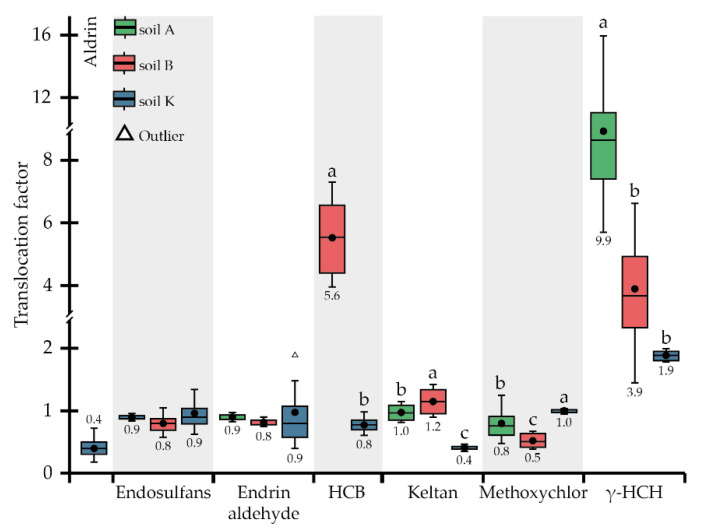
Translocation factors of OCPs. Different letters on the boxplots within one compound indicate a significant difference at *p* < 0.05.

Aldrin was detected in AGB and roots when the plant was grown in soil K, the most contaminated with OCPs, and in roots only when the plant was grown in the least contaminated soil A; this substance was not detected in the plant’s organs during growth in soil B (i.e., <LOD) (Table 2 and Table 3). A bioconcentration effect was observed only during development in soil A (*BCF* of 3.2), while in soil K, *BCF*s for AGB and roots were below 0.3 (Figure 1). Thus, aldrin was mainly accumulated in roots and not translocated to the AGB (Figure 2).

Endosulfans were found in the AGB and roots of plants grown in all research soils. The highest concentrations in plants were observed in soil A, followed by soils B and K, with 955, 664, and 520 μg kg^−1^ average concentrations within the plant, respectively, and all differences were significant (Table 3). Although no differences were observed between concentrations of endosulfans in AGB and roots, *BCF* values were inversely correlated with concentrations in soils, ranging from 11.5 (soil A) to 0.7 (soil K) (Figure 1). Despite the absence of a statistically significant difference between endosulfan concentrations in soils A and B (Table 2), the corresponding *BCF* values differed significantly.

Endrin aldehyde was accumulated in the plant’s organs for all research soils; the concentration of this substance in the plant tissues was higher for soil A (354 µg kg^−1^) compared to soil B (256 μg kg^−1^) and soil K (179 μg kg^−1^), and all pairs were significantly different. Similar to endosulfans, concentrations of endrin aldehyde in plant tissues were inversely correlated with concentrations in soils: *BCF* for AGB decreased from 5.4 (soil A) to 0.2 (soil K), and *BCF* for roots from 6.0 (soil A) to 0.2 (soil K), while OCP concentrations in soils were 62.8 (soil A), 130.8 (soil B), and 1088 µg kg^−1^ (soil K) (Figure 1; Table 2). Based on the *TLF*s (Figure 2), no actual translocation of endrin aldehyde from roots to AGB was observed, so the root system was the main accumulation organ.

HCB was quite strongly taken up by *P. tomentosa* during cultivation in all three soils studied (the concentration of this substance in plant tissues varied from 2.5 to 13.6 mg kg^−1^ (Table 3)); however, unexpectedly, HCB was not detected in the roots when the plant was grown in soil A (i.e., <LOD) (Table 3). The highest and lowest concentrations of OCP were detected in the AGB and roots of plants grown in soil B, most contaminated by HCB (41.6 μg kg^−1^) (Table 2). *BCF* values for HCB ranging from 58.9 to 571 (Figure 1) were much higher compared to other OCPs. Uptake of HCB depended on its concentrations in the soils: the average *BCF*s calculated for the whole plant in soils K and B were equal to 193 and 220, respectively, being not significantly different. In soil K, with the lowest concentration of HCB (14.0 μg kg^−1^), its accumulation in AGB and roots was not significantly different, with *BCF*s of 197 and 243, respectively. The HCB migration within the plant, represented by *TLF*s, was directly correlated with concentrations in the soils: the more OCP was present in the soil, the more enhanced migration into the AGB was observed (*TLF*s increased tremendously) (Figure 2).

Keltan was detected in both the AGB and roots of *P. tomentosa* during growth in all research soils. When the plant was developed in soils A and B, with relatively low concentrations of keltan (11.9 and 22.1 µg kg^−1^, consequently), the accumulation of OCP in the AGB and roots did not differ significantly between soils or plant organs (Table 3). In contrast, keltan concentrations in AGB (50.5 μg kg^−1^) and roots (121 μg kg^−1^) were significantly lower when plants grew in soil K and differed within plant organs (Table 3. The highest *BCF* of 17.0 was observed for soil A, followed by 8.7 and 2.6 for soils B and K, respectively (Figure 1). Accordingly, the uptake of keltan reduced with the increasing concentrations in soils. A similar trend was detected for *TLF* values (Figure 2).

Methoxychlor was accumulated almost equally in AGB and roots when the plants grew in the most (soil K) and least (soil A) contaminated soils (Table 3). In contrast, the concentrations of OCP in AGB (235 μg kg^−1^) and roots (464 μg kg^−1^) differed significantly when the plant was grown in soil B. *BCF* values decreased from 6.5 to 0.2 for AGB and from 8.5 to 0.2 for roots in soils A and K, respectively, representing the inverse correlation between the uptake of methoxychlor and its concentrations in the soils (Figure 1). The opposite trend was observed for the migration of OCP to AGB, i.e., the *TLF*s were 0.5, 0.8, and 1.0 for soils B, A, and K, respectively (Figure 2). Thus, the translocation occurred at relatively high concentrations in the soil (1307 μg kg^−1^).

The uptake of γ-HCH as well as endosulfans, endrin aldehyde, and keltan decreased with their increasing content in the soils studied (Table 1 and Table 3). For all soils, γ-HCH accumulation was higher in AGB than in roots; however, it was without a significant difference for soil K. *BCF*s for roots changed in a rather wide range (0.2–1.0), albeit not significantly different (Figure 1). *BCF*s for AGB decreased from 7.8 (soil A) to 3.5 (soil B) and 0.4 (soil K) in parallel with the increasing OCP concentrations in the soils. Translocation of γ-HCH was observed in all soils (Figure 2), and even at a sufficiently high concentration in the soil, OCP migration to AGB remained reasonable, with a *TLF* of 1.9, indicating the phytoextraction potential of *P. tomentosa* concerning this substance.

In addition to OCPs, research soils contained different TTEs; for some elements, the concentration in the soils exceeded the MPC (Table 2). Although As was detected in the three research soils, this element was not detected in the plant tissues (i.e., <LOD). Other TTEs originally presented in the research soils, i.e., Cr, Co, Ni, Cu, Zn, Cd, and Pb, were detected in *P. tomentosa* tissues (Table 3) at concentrations that varied significantly between soils (at least *p* < 0.05). The concentrations of Cr, Co, Ni, Cu, and Zn differed between roots and AGB as well (Table 3). The uptake of Cr, Cu, Zn, and Pb into the AGB correlated directly with their concentrations in the soils, while the accumulation in the roots showed the opposite trend. The uptake of Ni and Cd to both parts of *P. tomentosa* correlated inversely with their concentrations in the soils, while Co concentrations correlated directly (Table 3).

**Figure 3:**
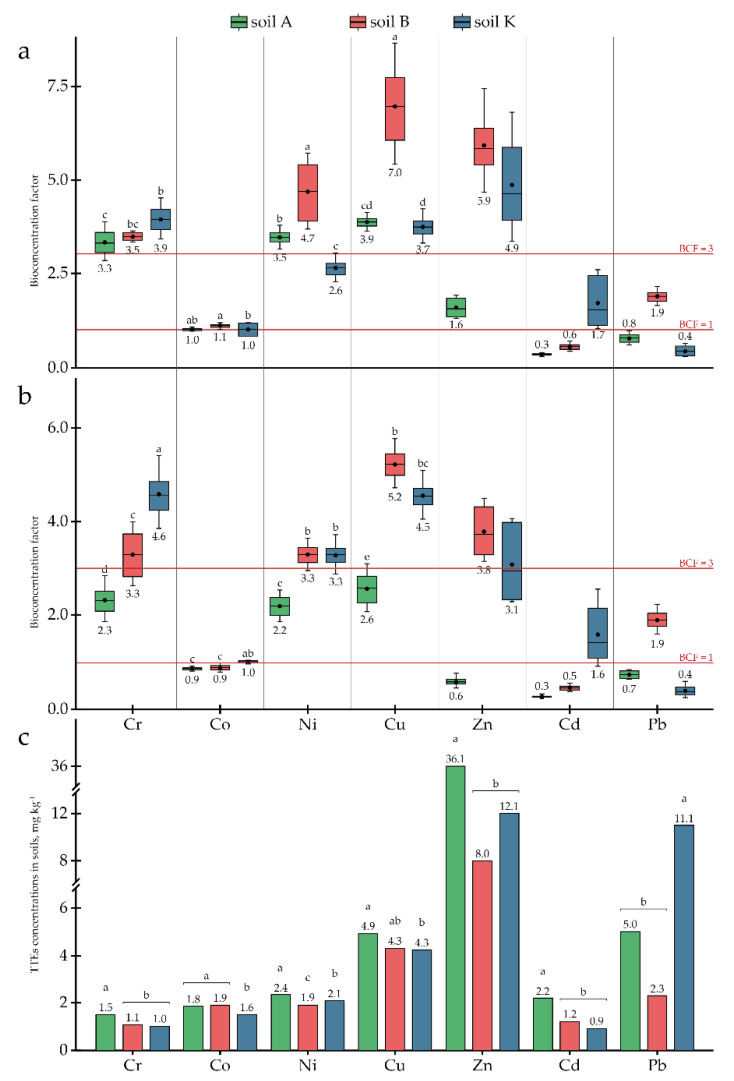
Bioconcentration factors for TTEs in plant parts: (**a**) AGB; (**b**) roots; (**c**) TTE concentrations in the research soils Different letters on the bar and boxplots within one element indicate a significant difference at *p* < 0.05.

**Figure 4:**
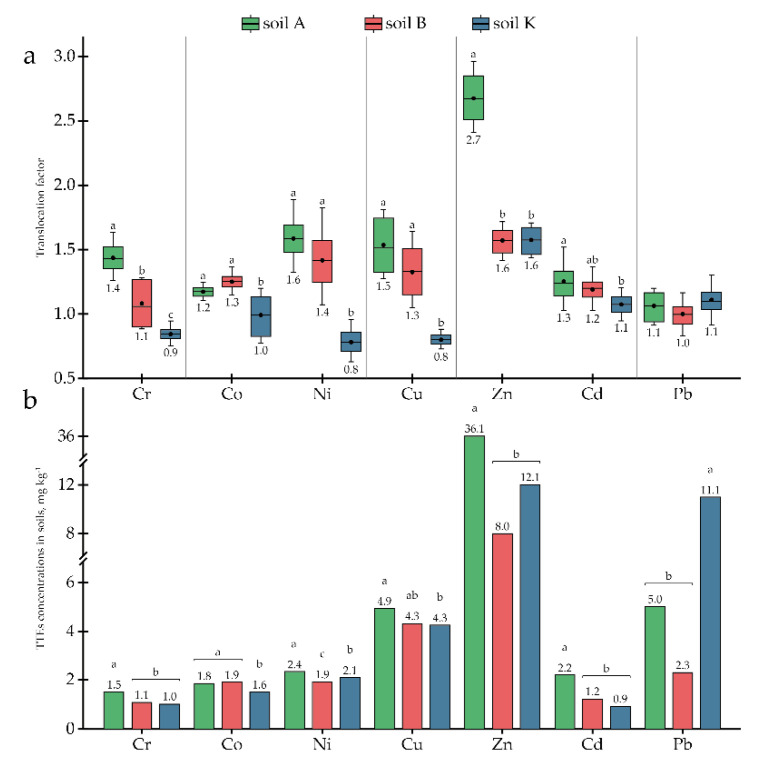
Translocation factors for TTEs: (**a**) *TLF*s; (**b**) concentrations in soil. Different letters on the bar and boxplots within one element indicate a significant difference at *p* < 0.05.

Since concentrations of Cu, Zn, and Cd in the research soils exceeded the MPC (Table 2), the potential of *P. tomentosa* to uptake and accumulate these elements was investigated in detail. The accumulation capacity of *P. tomentosa* concerning Cu ranged from 12.7 to 30.1 mg kg^−1^, depending on the soil. In soils A and B, Cu concentrations in the AGB were 19.1 and 30.1 mg kg^−1^, respectively, being significantly higher than in the roots (12.7 and 22.6 mg kg^−1^, respectively). In soil K, the Cu concentration was slightly higher in roots (19.4 mg kg^−1^) than in AGB (15.6 mg kg^−1^). The highest *BCF*s for AGB and roots were found in soil B with a medium Cu concentration (4.34 mg kg^−1^) (Figure 3). The *TLF*s correlated directly with the Cu concentrations in the soils (Figure 4).

The accumulation of Zn varied significantly in a range from 21.0 to 56.0 mg kg^−1^. Zn uptake to AGB was higher than uptake to roots in all research soils. *BCF*s for both AGB and roots correlated inversely with the element’s concentration in the soils, while the *TLF*s correlated directly (Figure 3 and Figure 4). However, in soil K, with an average concentration of Zn equal to 12.07 ± 3.26 mg kg^−1^, element uptake decreased and *TLF* remained at the same level. Furthermore, reduced uptake was observed in soil A, with high Zn contamination; however, translocation increased to 2.7.

Cd concentrations accumulated by *P. tomentosa* ranged from 0.53 to 1.29 mg kg^−1^, with almost equal distribution between plant parts. At the same time, plants showed an ability to bioconcentrate this element in soil K, with *BCF*s above 1 for both AGB and roots.

## 4. Discussion

The uptake and translocation of contaminants by *P. tomentosa* are highly variable and determined by their characteristics and the level of soil contamination. When analysing the phytoremediation process, some peculiarities were observed, such as the detection of heptachlor in the plants’ AGB in the absence of its traces in the roots. This observation can be explained by the rapid transformation of heptachlor in the living organisms into the more persistent and hazardous metabolite heptachlor epoxide [61], detected in the tissues of *P. tomentosa* (data not shown; soil B: 95.5 ± 13.5 µg kg^−1^ and 102 ± 3.3 µg kg^−1^ in AGB and roots, respectively). The absence of hexabromobenzene translocation to AGB could be due to its sufficiently high hydrophobicity coefficient (log *K_ow_* = 5.7) and low water solubility (0.16 µg L^−1^) [62]. The behaviour of plants concerning aldrin was quite different: in soil A, this substance was detected only in the roots of *P. tomentosa*; in soil B, it was not detected in any of the plant organs, and in soil K, aldrin was detected in both AGB and roots. Such behaviour could be explained by the rapid metabolisation of aldrin to less hydrophobic (log *K_ow_* = 5.4) dieldrin [63]. This assumption was confirmed by the presence of dieldrin in the AGB and roots of *P. tomentosa* when the plant was grown in soil K, despite this substance not being originally determined in the soil (Table 2).

Literature data on the phytoremediation potential of *P. tomentosa* toward OCPs are quite limited, as most researchers have mainly studied spiked soils with an essentially narrow range of OCPs, unlike the aged soils investigated in the current study. Therefore, the comparison of the phytoremediation potential of *P. tomentosa* was made with other phytoagents when at least one of the above criteria matched. In the earlier research, we studied the phytoremediation potential of *M. sinensis* while growing in soil K [52]. The comparison of results presented in [52] and obtained in the current study revealed that *M. sinensis* had a better ability to accumulate aldrin and γ-HCH compared to *P. tomentosa* (Table 3).

Results showed that *M. sinensis* accumulated aldrin only in the roots at a concentration of 308 μg kg^−^^1^, while *P. tomentosa* accumulated aldrin in the AGB (22.5 μg kg^−1^) and roots (57.5 μg kg^−1^) (Table 3). The accumulation of γ-HCH showed a reversed trend: *TLF* calculated for *M. sinensis* was 4.5 and thus 2.4 times higher than that of *P. tomentosa* (Figure 2). Rissato et al. [9] observed the uptake capacity of *Ricinus communis* L. in soil spiked with OCPs, most of which were present in the current research soils (A, B, and K). Although the uptake potential of *P. tomentosa* for aldrin, heptachlor, methoxychlor, and γ-HCH was higher than that of *R. communis*, the translocation of aldrin to AGB was lower. Sojinu et al. [64] studied the residues of 25 OCPs in 22 native plants, including energy crops (*Citrullus colocynthis, Manihot esculenta, Zea mays*, and *Pennisetum purpureum*). The phytoextraction ability of *P. tomentosa* related to aldrin, endosulfans, endrin aldehyde, heptachlor, methoxychlor, and γ-HCH when the plant was grown in soil A was compared with the phytoextraction ability of indigenous plants studied by Sojinu et al. [64]. The comparison showed that OCP concentrations were significantly higher in *P. tomentosa* AGB compared to the concentrations of the same substances in various plants investigated by Sojinu et al. [64].

In contrast to results obtained by Sojinu et al. [64], in the current study, aldrin and heptachlor were not detected in the AGB of *P. tomentosa*. The *BCF*s for AGB calculated from data presented by Sojinu et al. [64] showed that four energy crops were not able to bioconcentrate aldrin (*BCF*s ranged from 0.02 to 0.12). *P. tomentosa* showed more substantial phytoextraction potential for endosulfans, endrin aldehyde, methoxychlor, and γ-HCH, with *BCF* values of 10.8, 5.4, 6.5, and 7.8, respectively (Table 4).

There are different ways to characterise the ability of plants to uptake elements. Peterson [65] proposed two types of accumulation: the accumulation of an element to concentrations higher than in the growth medium (generally soil) or the possession of more significant quantities of an element than usual for such organisms (needing a reference concentration for the same plant). This concept is mainly valid for TTEs accumulation; however, it can also be extended to the accumulation of organic compounds.

Baker et al. [66] defined hyperaccumulation as when the concentrations of TTEs in the plant tissues surpass 0.01% for Cd; 0.1% for Co, Cu, Ni, and Pb; or 1% in the case of Mn and Zn. In the current research, the hyperaccumulation effect was not reached for any of the elements as their concentrations in plant tissues never exceeded 30 mg kg^−1^. The ability of *P. tomentosa* to bioconcentrate TTEs was variable: no real bioconcentration (i.e., *BCF*s around 1 or less) was noted for Co and very little for Cd and Pb (*BCF*s up to 2 only for slightly contaminated soils B and K). Significant bioconcentration was observed for Ni, Cr, and Zn (*BCF*s up to 4.7, 4.6, and 3.9, respectively), which became essential for Cu (*BCF*s up to 7.0). *BCF*s of several elements (Cr, Ni, Cd, and Pb) decreased more or less distinctly with their increasing concentrations in the soil, which seems to reflect a protection mechanism of *P. tomentosa*, which would limit the plant’s phytoremediation ability.

Translocation of TTEs from the roots to AGB of *P. tomentosa* was generally low (i.e., *TLF*s between 0.8 and 1.5). The observed high translocation of Zn in soil A could be linked to the higher concentration of the element in soil and its generally higher mobility [67]. This would lead to the preferable accumulation of Zn in plants’ AGB, especially when TTEs essential for plants are abundant in soil [67]. Indeed, Bahri et al. [39] observed the same peculiarity, with the increased translocation of Zn to the AGB of *P. tomentosa* grown in soil with higher Zn content.

Summarising the data presented in Figure 1, Figure 2, Figure 3 and Figure 4 and Table 3 it can be stated that *P. tomentosa* showed strong potential to accumulate certain soil-bound OCPs and TTEs, and can be proposed as an eligible species for phytoremediation programs for soils historically contaminated by a mixture of OCPs and TTEs.

## 5. Conclusions

The results of the current study indicate the successful cultivation of *P. tomentosa* during one growing season in soils historically contaminated with twenty OCPs and eight TTEs, collected in the vicinity of obsolete pesticide stockpiles in Talgar district, Almaty region, Kazakhstan. The phytoremediation potential of the plant was investigated in detail for OCPs, whose concentrations in the soils were significantly different (aldrin, endosulfans, endrin aldehyde, HCB, heptachlor, hexabromobenzene, keltan, methoxychlor, and γ-HCH), and for TTEs, whose concentrations were above the MPCs (Cu, Zn, and Cd). It was revealed that the potential of *P. tomentosa* to uptake OCPs and TTEs varied greatly depending on the type of contaminant and their concentration in the soils. Along with the ability to bioconcentrate Cr, Ni, and Cu, the phytoremediation potential of *P. tomentosa* to accumulate endosulfans, keltan, and methoxychlor provided very encouraging results. Moreover, the phytoextraction effect was found in the case of γ-HCH (*TLF*s of 1.9–9.9) and HCB (*BCF*s of 197–571). The *TLF* values for TTEs ranged from 0.8 to 1.5, indicating the low translocation of elements from the roots of *P. tomentosa* to AGB. Nevertheless, the observed high accumulation of Zn in the plant AGB can possibly be linked to the high mobility and concentration of this element in the soils studied. 

The results indicate the strong potential of the use of *P. tomentosa* in phytoremediation programs applied to soils contaminated with a mixture of organic and inorganic contaminants (OCPs and TTEs) with the simultaneous production of valuable biomass. A more extensive study is necessary to investigate the phytoremediation efficiency of *P. tomentosa* during multiyear vegetation at field scale, as the complexity of field conditions may modify the results that we obtained under greenhouse conditions.

## Figures and Tables

**Table 1:** Agrochemical profile of the research soils.

Parameter	Unit	Soil A	Soil B	Soil K
C	%	4.44 ± 0.11	5.27 ± 0.10	6.10 ± 0.02
pH (water)		7.48 ± 0.01	7.49 ± 0.02	7.85 ± 0.02
P_2_O_5_	mg kg^−1^	353 ± 15	71 ± 0	400 ± 5.0
K_2_O	mg kg^−1^	965 ± 15	740 ± 0	885 ± 25
Ca	meq/100 g	16.4 ± 0.50	19.2 ± 0	20.8 ± 0.70
Mg	meq/100 g	7.75 ± 1.40	5.05 ± 0.72	2.70 ± 1.23
Na	meq/100 g	0.16 ± 0	0.16 ± 0	0.38 ± 0.01
K	meq/100 g	1.31 ± 0	0.80 ± 0.03	1.04 ± 0.03

**Table 2:** Concentrations of OCPs and TTEs in research soils.

Contaminant	Pesticide Type ^a^	MPC ^b,c^	Soil A	Soil B	Soil K	*p*-Value	Root MSE
**OCPs, µg kg^−1^**							
Aldrin	I	2.5	12.2 **b**	96.0 **b**	345.2 **a**	**<0.01**	59.7
Chlordane	I	100	30.1	**<LOD**	72.1	0.34	47.1
Chlorobenzilate	I	20	277.6	5509	32,242	0.45	31,134
DDD	I	100	1153	2976	25,506	0.44	24,241
DDE	I	100	9709	69,847	777,967	0.40	716,310
DDT	I	100	1237	6274	10,023	0.33	6613
Dibutyl chlorendate	H	-	511.1	1285	2135	0.33	1208
Dieldrin	I	0.5	42.3	291.3	**<LOD**	0.18	185
Endosulfans	I	100	83.2 **b**	124.1 **b**	759.2 **a**	**<0.001**	63.0
Endosulfan sulfate	mI	-	654.5	265.7	356.0	0.46	373
Endrin	I	1	1289	181.3	44,085	0.41	42,462
Endrin aldehyde	mI	-	62.4 **b**	130.8 **ab**	1088 **a**	**<0.05**	394
HCB	F	500	21.3	41.6	14.0	0.07	11.7
Heptachlor	I	50	**<LOD**	118.4 **b**	269.0 **a**	**<0.001**	17.1
Heptachlorepoxide	I	50	190.3	**<LOD**	3029	0.39	3580
Hexabromobenzene	F	30	39.8 **c**	187.6 **b**	604.0 **a**	**<0.001**	54.0
Keltan (Dicofol)	I	100	11.9	22.1	32.9	**0.10**	10.7
Methoxychlor	I	1600	11.1 **c**	137.2 **b**	1307 **a**	**<0.001**	43.9
γ-HCH	I	100	19.3 **b**	20.1 **b**	76.4 **a**	**<0.001**	3.0
HCH isomers	mI	100	162.7	258.9	600.4	0.25	299.2
**TTEs, mg kg** ^**−1**^							
Cr		6	1.53 **a**	1.12 **b**	0.98 **b**	<0.01	0.12
Co		5	1.84 **a**	1.89 **a**	1.55 **b**	<0.001	0.06
Ni		4	2.36 **a**	1.85 **c**	2.08 **b**	<0.001	0.08
Cu		3	4.93 **a**	4.34 **ab**	4.28 **b**	<0.05	0.26
Zn		23	36.07 **a**	7.99 **b**	12.07 **b**	<0.001	4.15
As		2	0.32 **b**	0.67 **a**	0.27 **b**	<0.001	0.06
Cd		0.5	2.17 **a**	1.17 **b**	0.85 **b**	<0.001	0.23
Pb		32	5.01 **b**	2.25 **b**	11.11 **a**	<0.01	2.12

^a^ F: fungicide; I: insecticide; H: herbicide; mI: a metabolite of insecticide; ^b^ MPC values for OCPs as for the Republic of Kazakhstan [59]; ^c^ MPC values for TTEs, as for the Republic of Kazakhstan [60]; LOD = 0.1 µg kg^−1^. The concentrations of Cr, Co, Ni, Cu, and Zn reflect the mobile form, i.e., the fractions available to plants, while the concentrations of As, Cd, and Pb represent the total form. Means in the same line with different letters are significantly different.

**Table 3:** OCPs and TTEs concentrations in AGB (sum of leaves and stems) and roots of *P. tomentosa*. Different letters within one compound indicate a significant difference.

Contaminant	Soil A		Soil B		Soil K		*p*-Value			Root MSE
	AGB	Roots	AGB	Roots	AGB	Roots	SO Effect	PP Effect	Cumulative Effect	
HCB	12,170 **a**	**<LOD**	13,572 **a**	2449 **b**	2760 **b**	3395 **b**	<0.001	<0.001	<0.001	1679
Keltan	198 **a**	206 **a**	206 **a**	179 **a**	50.5 **c**	121 **b**	<0.001	0.11	<0.01	21.0
Methoxychlor	71.7 **c**	94.0 **c**	235 **b**	464 **a**	236 **b**	237 **b**	<0.001	<0.01	<0.01	47.8
γ-HCH	151 **a**	16.3 **c**	70.0 **b**	19.0 **c**	29.5 **bc**	15.5 **c**	<0.001	<0.001	<0.001	15.9
Cr	5.04 **a**	3.52 **c**	3.90 **bc**	3.69 **bc**	3.83 **bc**	4.45 **ab**	<0.10	<0.05	<0.001	0.33
Co	1.87 **ab**	1.60 **b**	2.10 **a**	1.67 **b**	1.56 **b**	1.57 **b**	<0.01	<0.01	<0.05	0.13
Ni	8.17 **ab**	5.18 **c**	8.65 **a**	6.08 **bc**	5.48 **c**	6.81 **abc**	<0.10	<0.01	<0.01	0.83
Cu	19.1 **bcd**	12.7 **d**	30.1 **a**	22.6 **b**	15.6 **cd**	19.4 **bc**	<0.001	<0.01	<0.01	2.39
Zn	56.0 **a**	21.0 **d**	46.6 **b**	29.7 **c**	55.8 **a**	35.4 **c**	<0.001	<0.001	<0.001	2.37
**Only SO Effect**										
Endosulfans	902	1007 **a**	588	739 **b**	493	546 **c**	<0.001	<0.05	0.61	83.5
Endrin aldehyde	336	372 **a**	231	281 **b**	159	199 **c**	<0.001	<0.10	0.96	42.9
Cd	0.74	0.60 **b**	0.63	0.53 **b**	1.29	1.20 **a**	<0.001	<0.01	0.72	0.08
Pb	3.87	3.65 **b**	4.24	4.26 **ab**	4.49	4.09 **a**	<0.05	0.25	0.59	0.34
**Not Available for Statistical Analysis**										
Aldrin	**<LOD**	39.0	**<LOD**	**<LOD**	22.5	57.5				
Heptachlor	**<LOD**	**<LOD**	331	**<LOD**	**<LOD**	**<LOD**				
Hexabromobenzene	**<LOD**	**<LOD**	**<LOD**	374	**<LOD**	**<LOD**				

Note: OCPs concentrations are presented in µg kg^−1^; TTEs concentrations are presented in mg kg^−1^; LOD = 0.1 µg kg^−1^. SO—soil origin; PP—plant parts.

**Table 4:** *BCF*s for OCPs accumulated in AGB of different energy plants.

Pollutant	Current Data	Data of Sojinu et al. [64]			
	*P. tomentosa*	*C. colocynthis*	*M. esculenta*	*Z. mays*	*P. purpureum*
Aldrin	ND	0.04	0.05	0.02	0.12
Endosulfans	**10.84**	0.38	0.99	0.57	3.46
Endrin aldehyde	**5.38**	0.51	ND	0.77	0.53
Heptachlor	ND	0.33	4.07	0.74	**19.95**
Methoxychlor	**6.46**	ND	0.70	0.51	0.38
γ-HCH	**7.82**	0.59	1.10	0.55	0.58

## Data Availability

Not applicable.
